# Update on the Pathogenic Implications and Clinical Potential of microRNAs in Cardiac Disease

**DOI:** 10.1155/2015/105620

**Published:** 2015-06-28

**Authors:** Mario Notari, Julián Pulecio, Ángel Raya

**Affiliations:** ^1^Center of Regenerative Medicine in Barcelona (CMRB), Barcelona Biomedical Research Park, Dr. Aiguader 88, 08003 Barcelona, Spain; ^2^Center for Networked Biomedical Research on Bioengineering, Biomaterials and Nanomedicine (CIBER-BBN), Spain; ^3^Control of Stem Cell Potency Group, Institute for Bioengineering of Catalonia (IBEC), Barcelona Science Park, Baldiri Reixac 15-21, 08028 Barcelona, Spain; ^4^Institució Catalana de Recerca i Estudis Avançats (ICREA), Spain

## Abstract

miRNAs, a unique class of endogenous noncoding RNAs, are highly conserved across species, repress gene translation upon binding to mRNA, and thereby influence many biological processes. As such, they have been recently recognized as regulators of virtually all aspects of cardiac biology, from the development and cell lineage specification of different cell populations within the heart to the survival of cardiomyocytes under stress conditions. Various miRNAs have been recently established as powerful mediators of distinctive aspects in many cardiac disorders. For instance, acute myocardial infarction induces cardiac tissue necrosis and apoptosis but also initiates a pathological remodelling response of the left ventricle that includes hypertrophic growth of cardiomyocytes and fibrotic deposition of extracellular matrix components. In this regard, recent findings place various miRNAs as unquestionable contributing factors in the pathogenesis of cardiac disorders, thus begging the question of whether miRNA modulation could become a novel strategy for clinical intervention. In the present review, we aim to expose the latest mechanistic concepts regarding miRNA function within the context of CVD and analyse the reported roles of specific miRNAs in the different stages of left ventricular remodelling as well as their potential use as a new class of disease-modifying clinical options.

## 1. Introduction

Heart or cardiac disease is an overreaching term used to describe a large family of conditions that affect the myocardial muscle. Disorders that fall under this category include blood vessel illnesses such as coronary artery disease, heart rhythm conditions termed arrhythmias, and, in general, all types of cardiomyopathies. The term cardiac disease is often incorrectly used interchangeably with the term cardiovascular disease (CVD). However, CVDs comprise a broad class of disorders that involve narrowed or blocked blood vessel(s) that can lead to myocardial infarction (MI) and heart failure (HF). In both China and the United States, CVDs were the major cause of morbidity and mortality in 2012 according to the World Health Organization [1]. Moreover, the patients that survive heart injuries experience, at best, progressive deterioration of their heart function unequivocally leading to HF [2]. Altogether, this information underscores the need to develop new therapeutic strategies potentially appointed to regenerate the damaged cardiac tissue.

Notably, a MI episode can lead to the annihilation within a few hours of about 25% of the 2–4 billion cardiomyocytes present in the human left ventricle [3]. The response to MI as well as to other forms of cardiac insults is described as left ventricular remodeling which involves all the structural, molecular, and functional adaptations of the heart in response to injury [2]. It is usually associated with increased volume and altered chamber configuration of the heart driven by a combination of pathological changes that comprise cardiomyocyte hypertrophy, myocyte apoptosis, myofibroblast proliferation, and interstitial fibrosis [2]. In particular, a MI involves acute oxygen deprivation to a portion of the ventricle leading to a confined area of ischemic cardiac cells, termed the infarct area. Given that adult cardiomyocytes have a very low intrinsic proliferation rate [4], vast replacement of damaged cardiomyocytes is precluded and a collagen-rich scar is formed instead. Hence, following an initial robust inflammatory response where dead cells are cleared by macrophage activity, cardiac fibroblasts infiltrate the damaged area to increase myocardial flexibility via deposition of extracellular matrix components [5], which marks the onset of the interstitial fibrosis process. While scarring culminates in a relatively quick preservation of myocardial integrity, it compromises the contractile activity of the heart permanently. Despite a wide range of available therapeutic options, the prevalence, mortality, and costs associated with CVDs continue to increase in the developed world as well as in the developing countries [6]. Current treatment options are limited to a surgical intervention to either restore patency or bypass affected coronary arteries, the application of passive cardiac support devices, and/or cardiac transplantation [6]. Although the ideal approach for HF patients would be to attempt myocyte replacement therapy with the objective of restoring cardiac muscle mass and function, current treatments and prospective research advances are geared towards the stimulation of the endogenous cardiac repair mechanisms [7–9]. Given the lack of a therapeutic approach to reverse the loss-of-functional myocardium, the development of efficient regenerative procedures is an urgent need in the field of modern cardiovascular research.

As an alternative, a promising cell-free strategy is based on the use of small molecules or paracrine factors to stimulate cardiomyocyte proliferation or differentiation of resident cardiac cells [10–12]. Recently, a new class of small noncoding RNAs or microRNAs (miRNAs) were identified as important posttranscriptional inhibitors of gene expression through their ability to block the translation of messenger RNA (mRNA) [13]. Hence, depending upon the presence and expression of a specific miRNA and/or its target as well as the physiological state of a cell, a miRNA can act as a “fine tuner” or even as an on/off switch of gene expression [13]. In this context, changes in miRNA levels as they relate to murine and human heart disorders and their implications in the development of different CVDs have been recently recognized; thus, certain miRNA expression signature patterns were shown to correlate with HF and cardiac hypertrophy [14]. To date, various studies have illustrated changes in miRNAs expression in a variety of human heart conditions such as MI [15], cardiac hypertrophy and fibrosis [16], and developmental heart disease [17]. They showed that the expression of numerous miRNAs is altered in such pathological processes and that different types of heart disease are associated with distinct changes in miRNA expression [18]. Therefore, understanding the regulation of molecules of mRNA biogenesis in both stem and cardiac progenitor cells will lead to a better understanding of the pathophysiological development of CVDs as well as contributing towards the development of novel therapeutic targets that promote endogenous cardiovascular regeneration. Not surprisingly, research on miRNAs in relation to the physiology and pathology of CVDs has become a rapidly evolving field. Recently, several excellent reviews have analyzed the progress made in understanding the role of miRNA therapeutics within the CVDs field [19–21] and circulating biomarkers within the blood stream for early detection of specific CVDs pathologies [22]. In this review, we examine the latest progress regarding the roles of miRNAs in the left ventricle remodeling process, as well as their potential for therapeutic intervention. We will focus our attention on the mechanistic concepts of miRNA function within the context of CVD progression and consider the specific roles of representative miRNAs in the different phases of left ventricular remodeling.

## 2. The Biology of miRNA: Overview

**Figure 1:**
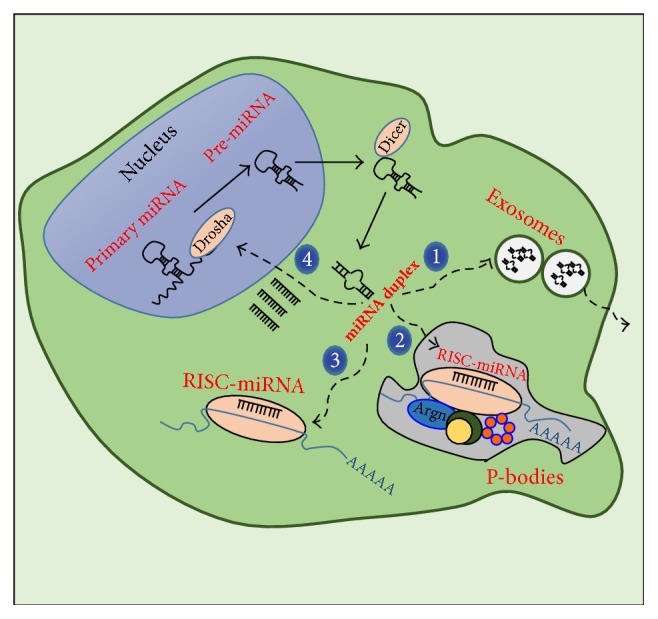
Representative scheme summarizing the biogenesis of the miRNA. The cartoon depicts the four main pathways followed by miRNA after extranuclear cleavage by DICER. The miRNA duplex can be processed to single mature miRNA and loaded into exosomes or microvesicles ready for extracellular release (1) or aggregates in P-bodies (2), a new model of mRNA regulation involving miRNA and other specific proteins [122]. However, miRNAs are preferentially loaded into the RISC complex in the cytoplasm, inducing silencing of target mRNA (3). Alternatively, miRNAs are able to enter back into the nucleus and directly bind the promoter region of target genes (4) and influence their expression. Argn = argonaute.

miRNAs are 21- to 23-nucleotide- (nt-) long noncoding RNAs able to modulate gene expression by targeting genes at the posttranscriptional level in a tissue-specific fashion. Thus, miRNAs bind mostly to the 3′ untranslated region of target genes and inhibit gene expression translationally and/or by destabilizing the target mRNA [13]. miRNAs are transcribed as regular genes from DNA by RNA polymerase II into primary miRNAs, which are processed to pre-miRNAs (70-nt stem loop oligonucleotides) in the nucleus by the Drosha complex (a type III RNase complex), then exported to the cytoplasm, and further cleaved by the DICER1 complex into 22-nt double stranded fragments [23] (miRNA duplex, Figure 1). At this point, miRNAs can be discharged to the extracellular space within vesicles (i.e., exosomes) responsible for miRNA-mediated cell-to-cell communication.* In vitro *experimentations proved that endothelial cell line culture overexpressing the transcription factor Kruppel-like factor 2 (KLN2) was able to generate extracellular vesicles highly enriched in miRNAs. KLN2 is an important transcription factor involved in atheroprotective stimuli and able to enhance the expression of a particular cluster of miRNAs [24]. Similarly, the finding of circulating miRNAs in different human fluids indicates a conceivable role of miRNAs as paracrine signaling mediators* in vivo,* suggesting a crucial role of miRNAs in cell-cell communication and paracrine signaling during either physiological or pathological processes [24, 25]. Of note, involvement of miRNAs/vesicle-mediated communication has been discovered between different populations of myocardial cells such as endothelial and smooth muscle cells (SMC) [24] and also between fibroblasts and cardiomyocytes as well [26]. This latter study demonstrated a crosstalk between cardiac fibroblast and cardiomyocytes through extracellular vesicles highly enriched in miR-21^*^, a documented inducer of cardiac hypertrophy [26]. Alternatively, one of the strands of the miRNA can be selectively loaded into the RISC complex, which is responsible for binding to target mRNAs and repressing their expression through degradation or translational inhibition (Figure 1). In particular, a model for cytoplasmic mRNA repression/decay regulation has been proposed [27], in which argonaute (an essential protein of the RISC complex) directly interacts with the P-bodies (cellular foci involved in mRNA turnover containing most of the proteins related with miRNA gene silencing) [28] (Figure 1). Additionally, some miRNAs are able to reenter the nucleus and directly bind to the promoter region of target genes to regulate their expression [29] (Figure 1).

Most miRNAs are predicted to target several mRNAs and most mRNAs have predicted binding sites for multiple miRNAs. In this manner, a single miRNA can affect an entire signaling pathway, converting them into appealing tools for cell-fate conversion approaches. Therefore, miRNAs perform diverse roles in many different cellular, developmental, and physiological processes [30, 31]. The relevance of the miRNAs as key players in phenotype specification mechanisms has been extensively confirmed by studies showing that the forced overexpression of miRNAs specifically upregulated in neuronal cells, embryonic stem cells, osteoblasts, blood progenitors, and cardiomyocyte stem cells, in specific culture conditions, was sufficient to induce the conversion of somatic cells towards each of these cell types [32, 33]. Hence, exogenous expression or inhibition of cardiac related miRNAs would presumably be a suitable tool for therapeutic applications in the context of CVD.

Relating to genomic organization, miRNAs are often clustered within miRNA families, comprising mature species similar enough to have evolved from a common ancestor and which are often regulated as a single cistron [34, 35]. This agrees with the notion that a family of miRNAs can affect a specific set of mRNAs in a redundant fashion, thereby significantly affecting their expression levels and hence the dynamics of any biological pathway.

A special mention in regards to the nomenclature of miRNAs, that is, the number assigned to a given primary miRNA, may not necessarily correspond to the same miRNA sequence across species. Moreover, the same miRNA sequence given or not the same nomenclature may not necessarily affect the same set of mRNAs in different species [35]. Hence, this lack of nomenclature consistency calls for caution when reading reports from research using different species' models.

## 3. miRNAs Implicated in Cardiac Development

**Figure 2:**
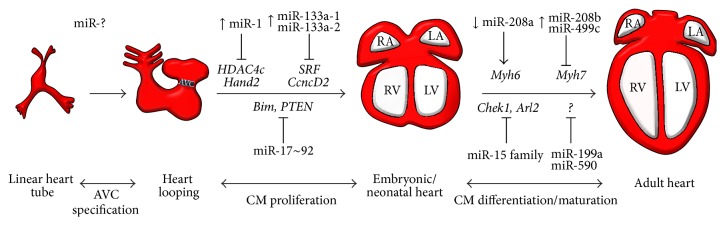
miRNAs control various stages of embryonic heart development. Schematic representation of the main stages of heart development. Murine cardiomyocyte progenitors become organized into a linear heart tube at day 7.5 post coitum (left). Subsequently, atrioventricular canal specification (AVC) as well as looping and swelling of the heart gives rise to the ventricular and atrial chambers. Between E13.5 and E15.5, the structure of the heart is complete and consists of the right atrium (RA), left atrium (LA), right ventricle (RV), and left ventricle (LV). miRNAs and their downstream targets are shown during embryonic heart development. Of note, compelling evidence for a critical role of miRNAs during AVC specification has been collected only in developing models such as zebrafish. It is however plausible that particular miRNAs also play an important role in AVC specification in mammals but has not yet been elucidated.

The first evidence for an essential contribution of miRNAs in heart development came from analysis of transgenic animals with conditional knockout of the miRNA processing enzyme DICER1 in cardiac progenitor cells (CPCs), defined as cells expressing early cardiac-specific genes such as* Nkx2.5* [36]. Mouse embryos lacking DICER1 in CPCs died before birth with severe cardiac defects such as pericardial edema and a poorly developed ventricular myocardium [37]. Interestingly, conditional knockout DICER1 in the adult mouse myocardium induced rapid biventricular enlargement, characterized by myocyte hypertrophy, sarcomeric disarray, ventricular fibrosis, and strong induction of the fetal gene program [38]. Moreover, miR-1 and miR-133, two specific and widely conserved miRNAs derived from the same precursor transcript, have been well established as fundamental factors in cardiac development [39, 40]. However, while miR-1 and miR-133 cluster on the same chromosomal locus and are transcribed together in a tissue-specific manner, they have distinct roles in modulating skeletal muscle proliferation and differentiation [40]. For instance, mice lacking the miR-1-2 genomic locus developed a range of abnormalities such as ventricular septal defects, cardiac arrhythmia, and myocyte hyperplasia with nuclear division of cardiomyocytes persisting postnatally [37]. It was shown that miR-1 promoted myogenesis by targeting histone deacetylase 4 (HDAC4), a nuclear factor that represses the transcription of muscle specification genes [40]. In addition, increasing miR-1 levels in the developing heart lead to a decreased pool of proliferating cardiomyocytes via the targeting of Hand2 and thereby controlling cardiomyocyte differentiation and proliferation during cardiogenesis [39] (Figure 2). In regards to miR-133, it was shown to promote mesoderm formation in mouse embryonic stem cells [41]. However, mice lacking either the mir-133a-1 or miR-133a-2 variants were normal, while about half of double-mutant mice displayed lethal ventricular-septal defects already at 1 day postnatally (p1) and those that survived to adulthood developed signs of dilated cardiomyopathy and heart failure at around 4 months of age. These phenotypes were linked to deregulation of miR-133a-1 targets such as cyclin D2 and serum responsive factors (SRF) [42] (Figure 2). Interestingly, targeting EGFR expression via miR-133a-1 overexpression in human mesenchymal stem cells (MSCs) promoted cardiogenic differentiation [43]. Recently, another muscle-specific miRNA was shown to favor cardiac lineage differentiation of human CPC and rat MSCs. In fact, miR-499 translocated from differentiated myocytes to CPCs via gap junctions promoting the differentiation of the latter into functionally differentiated cardiomyocytes [44], highlighting the role of miRNAs as paracrine factors that can influence gene expression and consequently cell behavior (Figure 2). This is of particular interest for the design of therapeutic strategies involving miRNAs as modulating agents considering that they are unusually stable in plasma and resistant to harsh conditions such as pH variation and high temperature [45]. Moreover, a 3′ mutation in the human miR-499 sequence altered the expression of different cardiac-specific mRNAs [46]. In a transgenic mouse model, it was similarly shown that increasing miR-499 expression resulted in myocardial hypertrophy and severe cardiac dysfunction by affecting the expression of early stress (i.e.,* Erg1*,* Erg2*) and structural (i.e.,* Myh7b*,* Acta1*) genes thereby magnifying the cardiac response to stress [47].

Finally, the miR-17~92 family, a well-known mRNAs cluster involved in cancer biology named OncomiR-1, consists of 6 different miRNAs all being described in different embryonic stages of heart development [48]. Data analysis from loss-of-function experimentations has suggested a specific role of the miR-17~92 cluster in ventricular septa specification as well as lung hypoplasia [48]. More importantly, the miR-17~92 family has emerged as important regulator of myocardial differentiation in particular in the specification of CPC in the second heart field which is required for normal outflow tract development [49] (Figure 2). Wang et al. demonstrated that the bone morphogenetic protein (BMP) signaling pathway triggers the transcription of several members of the miR-17~92 family which in turn repress the expression of cardiac progenitors genes such as* Isl-1 *(ISL LIM homeobox 1)and* Tbx1 *(T-box 1) [49].

At present, only scattered information is available on the expression of miRNAs in CPCs. In particular, a member of the miR-17~92 cluster, miR-17, was identified as the most differentially expressed miRNAs between neonatal and adult murine heart [50]. Preliminary attempts at manipulating CPCs behavior for stem cell therapy by altering miRNAs expression have met with limited success [44, 51]. However, an important role of miRNAs to improve homing, integration, and survival of CPCs engrafted in the infarcted myocardium has been described, and a mixture of miR-21, miR-24, and miR-221 is found to enhance the viability and survival of Sca-1^+^ CPCs both* in vitro* and* in vivo* [52].

## 4. microRNAs and Myocardial Regeneration

**Figure 3:**
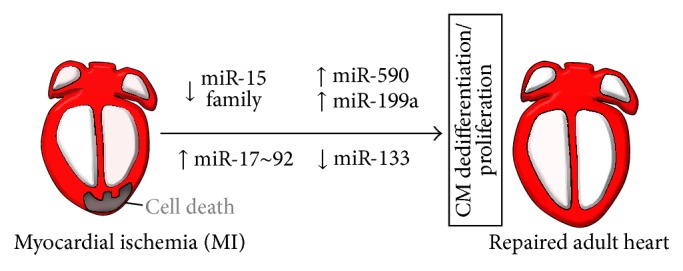
miRNAs can drive cardiac regeneration. Schematic representation of various miRNAs whose expression has been linked with the induction of cell cycle reentry mitosis and adult cardiomyocytes proliferation. Following myocardial infarction (MI), miRNAs that have been implicated in these processes are shown.

Several reports support the notion that microRNAs play an important role in myocardial regeneration. Hence, an alternative option for restoring the number of loss myocytes in CVD patients may be through activation of endogenous mechanisms for cardiomyocyte proliferation, thereby increasing myocardial muscle mass. In order to achieve myocardial regeneration and push the development of efficient therapeutic options, it is essential to uncover molecular mechanisms that will support cardiac regeneration. By definition, regeneration is a complex biological process by which animals can restore the shape, structure, and function of body parts lost after injury or experimental amputation. For instance, a number of lower vertebrates such as the newt and axolotl are able to renew parts of their body and even entire organs during their lifetime [53]. In particular, adult zebrafish show a conspicuous capacity to regenerate large portions of their heart after partial resection [54–56]. Notably, zebrafish heart regeneration after partial amputation of the ventricular apex proceeds by* de novo* formation of myocardial tissue that is indistinguishable from the surrounding myocardium both functionally and histologically [57]. Moreover, in this model, cardiomyocytes produced during regeneration are unlikely to be derived from undifferentiated stem or progenitor cells. Rather, the source of regenerating tissue is preexisting cardiomyocytes that after dedifferentiation reenter the cell cycle in response to injury [58]. It was recently shown that many miRNAs display compelling changes and are involved in fundamental regenerative signaling during zebrafish fin or organ regeneration [59]. Interestingly, a recent study purports that the neonatal mouse heart is also capable of complete regeneration following amputation of the apex or ischemic injury [60]. Hence, several groups have begun to investigate the involvement of microRNAs within the realms of the regeneration process using ventricular apex resection of the neonatal mouse heart, hoping this should be an ideal model to identify the miRNA profile regulating dedifferentiation and proliferation in the mammalian heart [61]. Although the mechanisms remain largely unknown, in zebrafish it was shown that miRNAs play a critical role in tissue regeneration [59]. For example, miR-133 was shown to promote cardiac regeneration in zebrafish [62], whereas miR-138 was associated with specification of the atrioventricular canal (AVC) which eventually gives rise to the cardiac valves indispensable for the unidirectional blood flow within the heart [63]. These observations prompted studies to ascertain whether similar mechanisms are present in the mammalian heart and, if so, whether experimental manipulation of miRNAs might trigger heart regeneration in mammals. More recently, Porrello and coworkers have established a model of ischemic MI in postnatal mice [64] and demonstrated that the neonatal heart can mount a regenerative response leading to cardiomyocyte proliferation of preexisting cardiomyocytes and a functional recovery in 21 days [60]. Interestingly, these authors also demonstrated that inhibition of the miR-15 family increased cardiomyocyte proliferation and improved ventricular systolic function after induction of MI in adult mice [64] (Figure 3). Members of the miR-15 family, including miR-15a, miR-15b, miR-16-1, miR-16-2, miR-195 and miR-497, have been implicated in cell cycle arrest and cell survival in different cell lineages via the regulation of many antiapoptotic genes and cell cycle inhibiting factors [65]. Members of this family are upregulated in the heart in response to stress conditions, MI, and cardiomyocyte death [16]. The multiplicity of miR-15 family members exemplifies one of the challenges associated with miRNA inhibition as a therapeutic strategy, as sequence divergence among different members of miRNA families prevents their collective inhibition by the delivery of a single antisense oligonucleotide inhibitor (antagomiR). Recent work performed using a model of ischemic and reperfusion in mice showed that inhibition of the miR-15 family reduced infarct size and improved cardiac function two weeks after delivering the injury [66]. Moreover, forced expression of miR-195 in heart was sufficient to cause cardiomyocyte loss leading to HF, and precocious activation of miR-195 in the heart suppressed the neonatal regeneration capacity of the murine heart [64]. The inhibitory influence of miR-195 on heart muscle regeneration appears to be attributable to the inhibition of a cohort of proliferative proteins and cell cycle inhibitors [64]. Hence, antagomiR-mediated inhibition of miR-15 family members represents an intriguing strategy to enhance cardiac repair following injury.

More recently, Senyo et al. found that a population of mononucleated proliferating cardiomyocytes appeared in the peri-infarct region in adult mice subjected to experimental MI, raising the rate of proliferating cardiomyocytes over the observed basal levels [67]. Interestingly, they also identified hypertrophic cardiomyocytes undergoing rounds of karyokinesis without mitosis through the measurement of DNA content, suggesting that at least two independent populations of cardiomyocytes with different proliferative capacities exist [67]. Although the response observed is insufficient to regenerate the damaged heart, the fact that the adult mammalian heart showed a cardiomyocyte-mediated regenerative response is a remarkable finding with exciting implications such as those related to miRNA manipulation to enhance this endogenous regenerative reaction. In fact, a report by Eulalio and colleagues had suggested that it is indeed possible to experimentally drive adult mammalian cardiomyocytes toward a proliferative state by manipulating miRNA expression [68]. The study focused on finding miRNAs that promoted the expression of proliferative markers. Using a library of 875 human miRNA mimics in a high-throughput screening approach, the authors found 204 miRNAs that induced greater than 2-fold induction of the proliferation rate of both rat and mouse cardiomyocytes and demonstrated that forced miR-199a and miR-590 expression with adenoassociated viruses increased the number of proliferating cardiomyocytes leading to a significant improvement of cardiac function after MI [68] (Figure 3). Other miRNAs that significantly increased postnatal cardiomyocyte proliferation in 7-day old mice were miR-590-3p, miR-199-3p, miR-33b, and miR-1825 [68]. miR-33b was previously shown to play a role in regulating cell proliferation and fatty acid metabolism [69, 70]. A recent study indicated that the miR-199a-204 cluster was involved in HF by facilitating a maladaptive metabolic shift to increased glucose metabolism rather than fatty acid utilization, as it would be normally found in the healthy myocardium [71]. Using a cardiac disease mouse model with transverse aortic constriction pressure overload, the authors found that mice treated with antagomirs of miR-199a and miR-214 displayed improved cardiac function as well as a normal arrangement of cardiomyocytes, with fibrosis and hypertrophy being significantly reduced compared to vehicle-treated control hearts [71]. Mechanistically both miR-199a and miR-214 directly repressed peroxisome proliferator-activated receptor delta (PPAR-*δ*), a critical regulator of mitochondrial fatty acid metabolism in the heart, but did not alter the expression of genes involved in glucose metabolism [71]. Another group reported that loss of function of the miR-17~92 cluster in mice resulted in abnormal myocardial differentiation deriving from second heart field cardiac progenitors, through repression of the* lsl-1* gene during embryonic cardiac development [49], suggesting a specific role of this miRNA cluster in the differentiation towards cardiac lineages during embryogenesis. The miR-17~92 cluster encodes six polycistronic miRNAs such as miR-17, miR-18a, miR-19a, miR-19b, miR-20a, and miR-92b of which not all have the same seed site. A more recent study reported that the miR-17~92 cluster, particularly miR-19a and miR-19b, may induce proliferation of cardiomyocytes and help protect the heart from ischemic injury caused by MI [72]. Compared with controls, proliferation of cardiomyocytes was decreased in miR-17-92 loss-of-function hearts and increased in miR-17~92 gain-of-function hearts. Importantly, after MI, miR-17~92 gain-of-function hearts had improved cardiac function, reduced scar size, and increased numbers of proliferating cardiomyocytes at the injury border zone, suggesting a crucial role in cardiac regeneration. Further,* in vitro* studies indicated that the miR-17-92 cluster induced cardiomyocyte proliferation through direct repression of PTEN by miR-19a and miR-19b, making this cluster also an attractive therapeutic option [73] (Figure 2).

## 5. miRNAs and CVDs

In 2007, Ikeda and colleagues were among the first to analyze microRNA expression by genome-wide profiling of human hearts with several conditions. They measured the expression of 428 miRNAs in 67 human left ventricular postmortem samples belonging to ischemic cardiomyopathy, dilated cardiomyopathy, and aortic stenosis cases (Table 1). Recently, several studies have now compared genome-wide miRNA expression profiles between normal cardiac tissue and those derived from mice or human patients with an array of different CVDs [16, 18, 74–76]. Table 1 summarizes the data from these studies highlighting the miRNAs that were found to be most deregulated in CVD tissue as compared to the control samples tested. Interestingly, the 18 miRNAs listed in bold, alone, account for almost 90% of all miRNAs expressed within the heart [75]. It is also worth noting that the deregulation of these miRNAs in CVD hearts did not seem to have an effect on other tissues, despite being ubiquitously expressed and playing important roles in various organ systems. For instance, miR-125a and miR-125b, which typically display a dramatic alteration in their expression after cardiac stresses [14, 74], are also fundamental regulators of hematopoietic stem cell differentiation and maintenance [77]. Therefore, modulating the expression of these miRNAs could potentially result in unwanted side effects in additional off-target organs, which calls for extra caution when thinking about future treatment strategies. Collectively, these expression-profiling studies also revealed distinct miRNA signatures for heart diseases of different etiologies, supporting the clinical applicability of miRNAs not only as therapeutic targets but also as disease biomarkers.

*Myocardial Infarction (MI)*. The first consequence of an event causing insufficient blood supply to the myocardium, that is, MI, is cardiac tissue necrosis and myocardial inflammation which leads, inexorably, to left ventricular pathological remodeling and dysfunction [78]. It has been well established that acute MI is associated with the deregulation of multiple genes and it is therefore reasonable for hypothesizing that miRNAs may be modulated and play a crucial role during an episode of MI. Many molecular targets investigated for their ability to limit ischemic cell death have been particularly focused on the mitochondrial permeability transition pore (MPTP) and oxidative stress pathways. In fact, MPTP opening is triggered by Ca^2+^ accumulation and also by various other stress conditions and plays a central role during myocardial necrosis [79]. A deeper understanding of miRNA involvement in these defensive mechanisms may provide new therapeutic targets to reduce cell death following MI.

It is well known that the Ca^2+^ ion is central for the cardiac contraction system and for the signaling networks that regulate pathological cardiac growth and remodeling. Intracellular Ca^2+^ overload can occur in cardiomyocytes as a consequence of ischemic injury or other stresses, leading to contractile dysfunction and ultimately cell death via apoptosis or necrosis through activation of proapoptotic Bcl-2 family members and opening of the MPTP [80]. Recently, Aurora and colleagues elegantly demonstrated that miR-214 plays a protective role against I/R damage by attenuating Ca^2+^ overload-induced cardiomyocyte death [81]. Thus, miR-214 transgenic mice were sensitized to I/R injury as evidenced by increased cardiac apoptosis following MI and I/R injury. From a mechanistic point of view, miR-214 deletion promoted the transcription of Ncx1 protein immediately following the ischemic events, the level of which continued rising 7 days after the injury; conversely, miR-214 downregulation of Ncx1 was sufficient to reestablish Ca^2+^ homeostasis in cardiomyocytes* in vitro* [81]. It has been proposed that the upregulation of miR-214 expression, in response to ischemic injury, protects myocytes from damage by attenuating Ncx1 levels in order to prevent excessive Ca^2+^ influx into the cytoplasm [81].

Another miRNA that has been associated with different types of CVDs and disease models generated from acute cardiomyocyte death is miR-126. Levels of miR-126 were increased in the noninfarcted zone at 6 h after coronary artery occlusion and at 24 h following a 30-minute ischemic insult [82]. miR-126 is encoded within one of the* EGFR7* gene introns and it was found to be highly expressed in both heart and lung tissues. Moreover, it has been implicated in the proangiogenic action of VEGF and FGF through the repression of Sprouty-related-protein-1 (Spred-1), an intracellular inhibitor of VEGF [83]. Interestingly, the detection of miR-126 within the apoptotic bodies of dying endothelial cells was shown to be involved in the paracrine signaling that repressed the function of regulator of G protein signaling 16 (RSG16), an inhibitor of G-proteins coupled with receptor signaling implicated in blocking apoptosis [84]. These studies suggest that pharmacological intervention on miR-126 expression may be a viable therapeutic strategy to enhance neoangiogenesis and cardiac repair in the ischemic myocardium.

**Figure 4:**
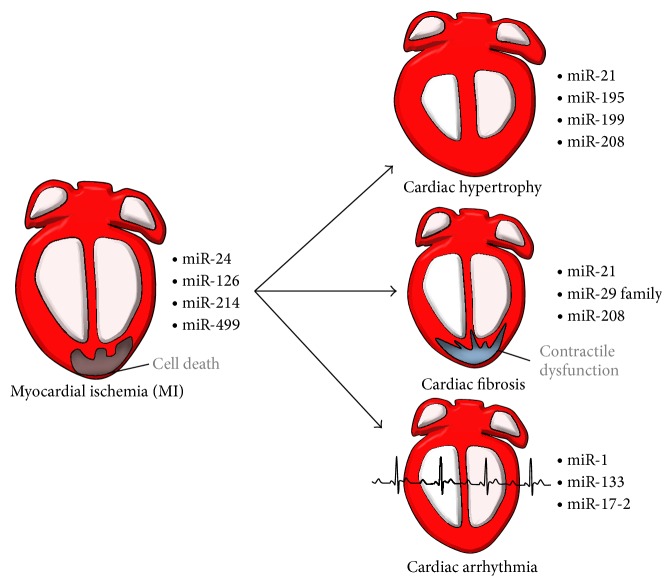
Schematic representation of the most relevant miRNAs reported during the different phases of left ventricular remodeling process.

Modulation of miR-499 levels attenuated cardiomyocyte death and the severity of MI induced by I/R [85]. This protective mechanism was based upon the inhibition of the acute apoptotic response of the cardiomyocyte. Thus, Wang and colleagues showed that the *α* and *β* isoforms of the calcineurin catalytic subunit, an important enzyme for cardiomyocyte metabolism, are a specific target of miR-499 in mice [85]. Calcineurin plays an important role in inhibiting cardiomyocyte apoptosis through the dephosphorylation of dynamin-related protein-1 (Drp1), an effector of the activated apoptotic pathway; this decreases Drp1 accumulation on the mitochondrial membrane preventing oxidative stress-driven cell death. Furthermore, miR-24 expression was also shown to be downregulated in the ischemic border but not in the remote zone of the myocardium of the left ventricle a few hours after induced-MI in rat myocytes [86]. It was speculated that miR-24 suppressed cardiomyocyte apoptosis partially by repressing the expression of the proapoptotic protein Bim [86]. Accordingly, poor blood oxygenation during MI induced the expression of the HIF-1*α* protein that, in turn, increased endogenous levels of miR-24, protecting cardiomyocytes from oxygen deprivation in a rat MI model [85]. Moreover,* in vivo* miR-24 overexpression in a mouse MI model also inhibited cardiomyocyte apoptosis, decreased infarct size, and improved cardiac function, rendering this miRNA an excellent target to impede oxygen deprivation-driven apoptosis [86] (Figure 4).

*Hypertrophy and Fibrosis*. The initial post-MI phase of left ventricular remodeling involves fibrotic repair of the necrotic area associated with scar formation and elongation of resident cardiomyocytes associated thinning of the ventricular walls [2]. Consequently, there is a left ventricular volume increase related to stroke volume augmentation and maintenance of normal cardiac output [2]. However, the remodeling process is driven mainly by hypertrophic cardiomyocyte elongation with the resulting wall mass increase and chamber enlargement, as well as a shift from an elliptical to a more spherical ventricular chamber configuration [87].

By using experimental models of remodeling rather than that of acute MI cell death, several groups demonstrated that miR-21 is one of the most deregulated miRNAs following I/R injuries (Table 1), despite the fact of being weakly expressed in normal cardiac tissue. For instance, Roy and colleagues reported an upregulation of miR-21 expression 2 and 7 days after MI induction in mice. Moreover, miR-21 inhibition increased the expression of the metalloproteinase-2 (MMP-2) via suppression of the PTEN pathway in cardiac fibroblasts [88]. This is particularly interesting, given that MMP-2 is an enzyme involved in the breakdown of extracellular matrix during tissue remodeling [89] with a potential therapeutic use in controlling scar formation. However, the role of this miRNA in CVDs is still controversial. For example, in 2008 Thum and colleagues reported that altering miR-21 expression* in vivo* increased cardiomyocyte hypertrophy by indirectly affecting cardiac fibroblast behavior [90] and this was subsequently confirmed by miR-21 overexpression in various* in vitro* systems [74]. Conversely, other studies reported miR-21 to have an antihypertrophic effect on isolated cardiomyocytes* in vitro* [91], to reduce infarct size following I/R injury, and to support cellular outgrowths on cardiomyocytes [92] or even an antiapoptotic effect on isolated cardiomyocytes from transgenic mice [93]. The reasons for such inconsistencies among those studies are unclear. It is possible that the biological consequences of miRNA-21 enrichment during CVDs are in part due to an increase in cardiac fibroblast cell number rather than from a direct effect of the injury itself, given that miR-21 is abundantly expressed in cardiac fibroblasts but not cardiomyocytes [17]. Moreover, miR-21 is expressed in many other tissues, including vasculature, hence precluding the discrimination between primary and secondary cardiac effects in models that use ubiquitous downregulation of miR-21 such as intravenous injections of antagomirs. This is supported by the fact that cardiomyocyte-specific overexpression of miR-21* in vivo* does not induce a distinct cardiomyocyte phenotype [90]. Given that the effects of miR-21 modulation during MI* in vivo* and on isolated cardiomyocytes* in vitro* are not well understood, it is currently unclear whether miR-21 holds a value as a prospective therapeutic tool.

Van Rooij and colleagues described a deregulation of specific cardiac miRNAs during MI induced by a model based upon occlusion of the left coronary artery in mice. Among the miRNAs modulated in this system, all three members of the miR-29 family were downregulated. These results were validated by real-time PCR analysis and confirmed using human patient samples (Table 1) [16]. Interestingly, the miR-29 family targets a pool of mRNAs that encode proteins involved in fibrosis such as collagens, fibrillins, and elastin [92]. Thus, downregulation of miR-29 would predictively increase the expression of these mRNAs and enhance the fibrotic response. Indeed, downregulation of miR-29 with antagomirs induces the expression of collagens both* in vitro* and* in vivo*, whereas overexpression of miR-29 in fibroblasts reduces extra cellular matrix deposition [16]. These data were confirmed in bleomycin-induced fibrosis in the lung where the expression on miR-21 inversely correlated with the expression levels of profibrotic target genes and the severity of the fibrosis [94]. Thus, the miR-29 family acts as a regulator of cardiac fibrosis and represents a potential therapeutic target to control tissue fibrosis in general.

Also worth noting is the activity of miR-208 during heart fibrosis. This is a cardiac-specific miRNA encoded by an intron of the* MYH6* gene coding for *α*-myosin heavy chain (*α*-MHC), the major contractile protein expressed in rodent adult hearts [95]. In contrast, both miR-208b and miR-499 are encoded by introns of* MYH7* that codes the *β* isoform of myosin heavy chain (*β*-MHC), the isoform expressed during the fetal period. Thyroid hormone (T3) stimulates the expression of *α*-MHC and consequently of miR-208 after birth while repressing the expression of the embryonic isoform of the gene (Figure 2). A hallmark of pathologic hypertrophy subsequent to MI is the reactivation of a set of fetal genes, including those encoding for atrial natriuretic peptide (ANP), *β*-type natriuretic peptide (BNP), *α*-skeletal actin, and *β*-MHC [96]. Downregulation of *α*-MHC and upregulation of *β*-MHC are common responses to cardiac injury regardless of species; however, most relevant to cardiac disease is the finding that miR-208a knockout mice display reduced fibrosis and hypertrophy in response to thoracic aortic banding, which induces cardiac hypertrophy by increased afterload on the heart and is accompanied by downregulation of *α*-MHC and upregulation of *β*-MHC [95]. These transgenic animals fail to upregulate MYH7 expression, which is a common marker for pathological cardiac remodeling. These findings support the notion that therapeutic inhibition of miR-208a could induce benefits in the setting of cardiac remodeling. Indeed, Montgomery et al. showed that administration of the antagomirs against miR-208a reduced fibrosis and inhibited the activation of* MYH7* transcription in transgenic mice susceptible to hypertension and heart failure [97]. These findings provided the first proof of concept for the therapeutic use of antimir-208a agents in a setting of CVDs. Unexpectedly, mice treated with antimir-208a displayed resistance to obesity induced by a high-fat diet [98] indicating multiple effects of miR-208 on unknown targets that need to be accurately evaluated before any treatment strategy is devised (Figure 4).

*Cardiac Arrhythmia*. Heart rhythm problems or cardiac arrhythmias occur when the electrical impulses that coordinate heart contraction malfunction cause the heart to beat too fast or too slow. In fact, the terms arrhythmia refers to any changes from the normal sequence of electrical impulses of the heart. The electrical impulse of the heart is generated by specialized cells in the sinoatrial node and propagates to the atrial and ventricular myocardium through a specialized conduction system, whereas coordinated depolarization of adjacent cells in the myocardium is accomplished via gap junctions [99]. Membrane excitability depends on the activity of ion channels and, in particular, those for Na^+^, Ca^2+^, and K^+^, which together with gap junction proteins such as connexin-43 are all critical regulators of cardiomyocyte polarization and depolarization during contraction and relaxation. It is important to note that fibrotic deposits and more in general scar tissue within the myocardium alter the normal electric conduction of the heart, generating profound changes in the normal electrical impulse. Several miRNAs, including miR-1 and miR-133, are predicted to target ion channel proteins and therefore play important roles in cardiac conduction and in the onset of arrhythmias during CVDs. Recently, using a combination of luciferase reporter assays and Western blotting, it was reported that gap junction protein a1 (GJA1) and the potassium inwardly rectifying channel member 2 (KCNJ2) are both specific targets of miR-1 in rats heart [100]. Moreover, miR-1 levels increased in individuals with coronary artery disease, and its overexpression in normal hearts resulted in slowed conduction velocity of the depolarization wave and prolonged repolarization, leading to the development of arrhythmias. Conversely, blocking miR-1 function with antisense oligo-miR-1 in rat infarcted hearts normalized the expression of connexin 43, thus reducing arrhythmias after MI [100]. In support of these findings, Zhao et al. described that mice knock-out for miR-1-2 has several ECG alterations such as a lower heart rate, shortened PR interval, and widened QRS and died as a result of cardiac arrhythmias [37], confirming the implication of miR-1 within the electric conduction system of the heart. Mechanistically, it has been suggested that at least part of the electrical alterations observed in knockout mice was due to the increased expression of Irx5 and Irx4, two transcription factors that regulate endogenous levels of Kcnd2, a K^+^ channel protein involved in cardiac repolarization [37] (Figure 4).

*Nonischemic Cardiomyopathy*. Literally, cardiomyopathy means “heart muscle disease” and it is defined as a progressive disorder of the myocardium associated with abnormalities in organ morphology and structure mainly due from MI events [101]. Cardiomyocyte death, cardiac fibrosis, and cardiomyocytes hypertrophy are all features of cardiomyopathies generated, usually, by ischemic insults. Nonischemic cardiomyopathies, instead, originate from congenital malformations of the heart's structure, such as alteration of the sarcomere complexes, cell-cell or cell-matrix junction dissolution, or electrical conduction abnormalities, but can also arise from various environmental factors such as hypertension, diabetes, or life style factors [99]. Among nonischemic cardiomyopathies, diabetes is an independent risk factor and a major cause of chronic cardiovascular complications [102]. About 80% of deaths associated with diabetes are due to heart complications in particular to diabetic cardiomyopathy [102]. Several recently published reviews outline important findings in the context of diabetic cardiomyopathy, regarding the putative mechanism of the disease progression in relation to the use of the miRNAs as potential diagnostic markers, as well as therapeutic targets [103–105]. Notably, Jaguszewski et al. identified four circulating miRNAs, namely, miR-1, miR-16, miR-26a, and miR-133a, as putative biomarkers for the differential diagnosis of two conditions that have until now been clinically indistinguishable [106].

## 6. miRNAs and Therapeutic Options

**Figure 5:**
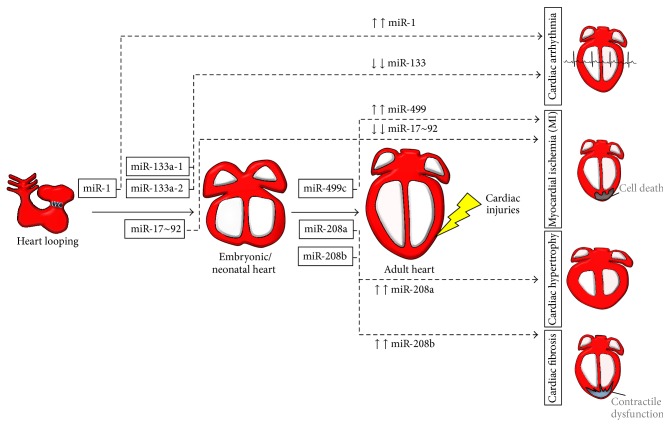
microRNA network in cardiac physiology and pathologies. The expression of miRNAs found of relevant importance in cardiac development (boxed miRNAs) is also involved in the remodeling process of the heart. Different modulation of miRNA expression enhances pathological outcomes at the myocardium and can be specifically targeted in a precise manner to improve the therapeutic applicability of miRNAs.

miRNAs may be of clinical value in the context of CVDs, both as therapeutic targets and as biomarkers to follow disease diagnosis and prognosis. The therapeutic potential of miRNAs in CVDs was first proposed in the light of results from animal studies that unveiled important roles of miRNAs in several contexts of cardiac development and disease. Since mice knockout for DICER1 shows phenotype in cardiomyocytes, smooth muscle cells, and endothelial cells leading to heart defects [107], it follows that reestablishing miRNA to normal levels could have a therapeutic effect on the context of cardiac disease. In principle, one of the advantages of the miRNAs as therapeutic tools in CVDs is their ability to target various factors in an established biological context. Because of the intrinsic nature of miRNAs, several mRNA targets may be downregulated by a single mature molecule, increasing the likelihood of affecting multiple components within a biological pathway. In addition, there are molecular tools that can affect simultaneously several miRNAs, thus further increasing the range of biological action. The design of specific inhibitory molecules, called miRNA sponges, may allow the inhibition of several miRNAs simultaneously, with a very high efficacy and the ability to specifically modify the dynamics of any pathway [108, 109]. In addition, inducing gain- or loss-of-function is technically more feasible, both* in vivo* and* in vitro*, using miRNAs rather than mRNA or DNAs, and miRNAs show higher stability [110]. Using a variety of animal models, it has been shown that inhibition of miRNAs upregulated upon cardiac malfunctioning by pharmaceutical or chemical molecules has reestablished normal cardiac function and, in some cases, even reduced the infarct size [65, 95, 96]. Considering this, therapeutic inhibition of miRNAs in CVDs is currently based on the fact that specific microRNAs have been investigated and found to be fundamental regulators in distinct phases of embryonic heart development and they were also described in the pathogenesis of different remodeling processes. Moreover, loss-of-function approaches could be designed using inhibitory tools such as miRNAs sponges to specifically block different miRNAs whose expression was related with different aspect of different CVDs in order to attenuate the response to cardiac injuries (Figure 5).

However, the therapeutic use of miRNAs faces a series of obstacles that should be taken into account and that hamper their current application on CVDs. The delivery of mature species mimicking miRNAs to induce gain of function or of chemically engineered molecules that specifically inhibit miRNA function should be accompanied by adjuvant strategies to address four outstanding issues: increasing tropism for the heart; blocking degradation by RNAse; reducing inflammatory response caused by naked nucleic acids; and achieving cytosolic delivery once it reaches the target organ. Delivery of small RNA molecules is often achieved by the means of liposomal or polymeric vehicles. Overall, these vehicles are able to protect the RNA species, increasing its stability and cellular uptake and strongly reducing any inflammatory response [111, 112]. However, delivery of these miRNA-containing vehicles to the heart is a random event, being the liver, spleen, and lungs the preferred organs that they reach [113, 114].

To circumvent the problem of targeting the heart in a specific-manner, different strategies have been devised ranging from direct intramyocardial injection which has been shown to reduce apoptosis and infarct size and to increase cardiomyocyte proliferation in infarcted rats [115] to the use of cardiac-specific antibodies that direct delivery vehicles to restricted cell populations. Nonetheless, in the case of CVDs, this last approach is still under debate because of the lack of reliable cardiac-specific antigens [116]. As an alternative, the use of delivery vectors traditionally used for long-term transgene expression can also be applied for miRNA gain or loss-of-function systems [68]. Primary miRNAs or sponge miRNAs can be engineered into adenoassociated viruses (AAV) to be expressed under strong promoters. The discovery of AAVs serotypes with increased cardiac tropism (AAV9), although in a nonspecific manner (it targets brain and liver as well), has created a window of opportunity for design clinically compatible strategies to inhibit or overexpress miRNAs in the future [117].

Regardless of these arguments, anti-miRNA therapeutics have recently turn into reality by the success of Phase I and Phase II clinical trials of Santaris Pharma antimir against miR-122,* miravisen*, which is currently in development as a cure for hepatitis C virus [118]. It is hoped that these promising findings will accelerate the development of miRNA-targeting therapeutics into additional disease areas such as that of CVD.

Finally, miRNAs can be used as well as disease biomarkers for the diagnosis and/or prognosis of various types of CVDs. The existence of circulating miRNAs in the blood stream of CVDs patients can be harnessed in the clinical setting as promising prognosis or diagnostic tools. We direct interested readers to excellent review by Creemers and colleagues on the different issue that need to be taken into account for the use of miRNAs as diagnostic tool and a comprehensive list of circulating miRNAs described in the context of CVDs [117].

## 7. Conclusion

In the present era of genomic discoveries, miRNAs represent a unique class of endogenous noncoding RNAs that are highly conserved across species and that repress gene translation upon binding to mRNA, thereby having a versatile influence on various biological processes [13]. This inhibition can have profound effects on cardiac function, fueling the excitement for future exploration of miRNAs as therapeutic tools. To date, various studies have pointed to the importance and authority of miRNAs in controlling different mechanisms associated with the biogenesis and establishment of CVDs. This important role makes them fascinating targets for therapeutic interventions. Nonetheless, it is important to note that different genome-wide miRNAs expression analysis studies have reported controversial results regarding the deregulation of specific miRNA expression in the context of the same CVDs (Table 1). Hence, numerous challenges and hurdles remain in the path towards the development of miRNA-based therapeutics. Firstly, in contrast to the situation described for miRNAs in lower vertebrates, the effects of individual miRNAs on mRNA and protein processing in mammals are relatively modest, as judged by the results of various loss-of-function studies [19]. Secondly, unlike conventional drug molecules, miRNAs can target many mRNAs at the same time and therefore influence the translation of multiple genes that contribute to common cellular function mechanisms and the development of pathological conditions [19]. However, a single miRNA will probably target unrelated genes and possibly produce undesired changes in gene expression. For example, antagomirs directed against the cardiac-specific miR-208a were shown to prevent over-weight and metabolic disorder in mice maintained on a high-fat diet [98], a side effect not expected to be based on the restricted expression pattern of miR-208a. Conversely, inhibitions of miR-29 were proved to be effective against cardiac fibrosis in two different organs such as heart and lung using a mouse model [94]. Most of the animal studies performed to date have focused on the phenotypic effects of miRNA inhibition in target organs and have largely overlooked off-targets effects that might be present in other tissues. Moreover, the doses used in most laboratory studies are unlikely to be feasible in a clinical setting. Follow-up preclinical studies will have to monitor appropriate dosing regimens in order to establish the lowest possible efficacious doses while attempting to prevent unwanted offside effects.

As an alternative to microRNA-based therapies, some investigators have turned their attention to direct lineage reprogramming conversion strategies, which involve switching one somatic cell type into another. The direct reprogramming of fibroblasts within the heart into functional cardiomyocytes using a combination of three or four factors (GATA4, Mef2C, Tbx5, and/or Hand2) is an exciting new therapeutic approach to treat CVDs and replace the damaged myocardium [119, 120]. Considering this, recent investigations suggested that miRNAs in collaboration with defined cardiac transcription factors might regulate directly a reprogramming process of a somatic cell type into another, adding a new exciting level for therapeutic intervention [121]. As biomarkers, instead, miRNAs have been convincingly shown to be an important tool to aid in early diagnosis of CVDs [117] and might have a brighter future as such clinical tools.

## Figures and Tables

**Table 1:** The literature review summary of miRNAs found to be deregulated in cardiovascular diseases. A number of studies have used high throughput miRNA profiling technologies to compare miRNA expression levels between control and affected tissues from cardiac disease inducing mice models or postmortem samples from failing human myocardium. The heading of the table identifies the specific study, followed by the species and diseases modeled, and the experimental design as well as the microarray technologies used to analyze the data. In bold, in rows below, the most deregulated miRNAs according to the different studies are displayed. The following miRNAs tabulated that are not in bold represent other cardiac specific miRNAs found to be deregulated in the five studies referenced but not directly mentioned in our review. Arrows illustrate the fold change of upregulation or downregulation of listed miRNAs as compared to control samples. miRNAs with the same seed region were combined into families. The family was labeled as regulated when at least 3 members of the family displayed a change in expression profile. Each family includes the following: let-7 family = let-7a, let-b, let-c, let-d, let-e, let-f, let-g, let-h, let-I, and let-j; miR-15 family = miR-15a, miR-15b, miR-16, miR-195, miR-424, and miR-497; miR-17 family = miR-17-5p, miR-20a, miR-20b, miR-93, miR-106a, and miR-106b; miR-29 family = miR-29a, miR-29b, and miR-29c; miR-30 family = miR-30a, miR-30b, miR-30c, miR-30d, and miR-30e. LAC = left coronary artery constriction; HF = heart failure; ICM = ischemic cardiomyopathy; DCM = dilated cardiomyopathy; d = days. ↑↑↑ ≥ 7.5; ↑↑ ≥ 2.5; ↑ ≥ 1 fold increase; —: not available; ↓ ≥ −1; ↓↓ ≥ −2.5; ↓↓↓ ≥ −7.5 fold decrease.

Study	van Rooij et al. [16]			Ikeda et al. [18]			Matkovich et al. [76]		Rao et al. [75]	Cheng et al. [74]		

Species	Mouse	Mouse	Human	Human	Human	Human	Human	Human	Mouse	Mouse		

Model of disease	LAC (3 days)	LAC (14 days)	HF	ICM	DCM	Aortic stenosis	DCM and ICM	HF	*Dgr8* conditional KO mouse	Thoracic aortic constriction		

Microarray platform	miRMAX microarray			Bead-based flow cytometric miRNA			Ncode microarray		Illumina microarray	mirVana microarray		

miR	LAC (3 d)	LAC (14 d)	HF	ICM	DCM	AS	DCM/ICM	HF	*Dgr8* KO mouse	TAC (7 d)	TAC (14 d)	TAC (21 d)

***Let-7 family***	—	↑	↑↑	↑	↑	↑	↑↑	↑↑	↑↑↑			
***15 family***	↑↑	↑↑	↑	↑	↑	↑	↑↑	↑↑		↑	↑	↑
***29 family***	↓↓	↓↓	↓↓				↑	↑	↑	↓	↓	↓
***30 family***	↓	↓↓	↓		↓	↓	↑↑	↑↑	↑	↓↓	↓↓	↓↓

***1***				↓	↓	↓	↑↑↑	↑↑	↑↑↑			
*21 *	↑↑	↑↑↑	↑↑				↑↑	↑		↑↑↑	↑↑↑	↑↑
***22***	↓↓	↓↓	↓↓				↑↑↑	↑↑	↑↑			
***23a, b***				↑	↑	↑	↑↑	↑	↑			
***24***				↑	↑	↑	↑↑	↑	↑			
***26a, b***				↑	↑	↑	↑↑	↑	↑			
***27a, b***				↑	↑	↑	↑↑↑	↑	↑	↑	↑	↑
***125a, b***				↑	↑	↑	↑↑	↑↑	↑			
***126***	↓↓↓	↓↓↓	↓	↑	↑	↑	↑↑	↑↑	↑↑	↓	—	↑
***133a, b***							↑	↑	↑↑	↓	↓	↓
***143***	—	↓↓	↓↓↓				↑	↑	↑↑			
***208***									↑↑			
*214 *	↑↑	↑↑	↑↑	↑	↑	↑				—	—	↑↑
***379***	↑	↑↑↑	↑									
***486***										↓↓	↓	↓
*499 *	↓	↓↓	—	↑	↑	↑	↑↑	↑	↑			
***638***	—	↑↑↑	↑↑				↑	↑				

*10a, b *	—	↑↑	↑↑			↓						
*146a, b *	↑↑	↑↑↑	↑									
*150 *	—	↓	↓	↑	↑	↑				↑↑	↑↑	↑↑
*223 *	↑↑↑	↑↑	↑									
*92 *	↑	↑	↑									
